# Mining Herbaria for Plant Pathogen Genomes: Back to the Future

**DOI:** 10.1371/journal.ppat.1004028

**Published:** 2014-04-24

**Authors:** Kentaro Yoshida, Hernán A. Burbano, Johannes Krause, Marco Thines, Detlef Weigel, Sophien Kamoun

**Affiliations:** 1 The Sainsbury Laboratory, Norwich Research Park, Norwich, United Kingdom; 2 Max Planck Institute for Developmental Biology, Tübingen, Germany; 3 Institute of Archaeological Sciences, University of Tübingen, Tübingen, Germany; 4 Biodiversity and Climate Research Centre (BiK-F), Frankfurt, Germany; 5 Institute of Ecology, Evolution and Diversity, Goethe University, Frankfurt, Germany; 6 Senckenberg Gesellschaft für Naturforschung, Frankfurt, Germany; 7 Centre for Integrative Fungal Research, Frankfurt, Germany; Duke University Medical Center, United States of America

Since the dawn of agriculture, plant pathogens and pests have been a scourge of humanity. Yet we have come a long way since the Romans attempted to mitigate the effects of plant disease by worshipping and honoring the god Robigus [1]. Books in the Middle Ages by Islamic and European scholars described various plant diseases and even proposed particular disease management strategies [1]. Surprisingly, the causes of plant diseases remained a matter of debate over a long period. It took Henri-Louis Duhamel du Monceau's elegant demonstration in his 1728 “Explication Physique” paper that a “contagious” fungus was responsible for a saffron crocus disease to usher in an era of documented scientific inquiry [2]. Confusion and debate about the exact nature of the causal agents of plant diseases continued until the 19th century, which not only saw the first detailed analyses of plant pathogens but also provided much-needed insight into the mechanisms of plant disease. An example of this is Anton de Bary's demonstration that a “fungus” is a cause, not a consequence, of plant disease [3]. This coming of age of plant pathology was timely. In the 19th century, severe plant disease epidemics hit Europe and caused economic and social upheaval. These epidemics were not only widely covered in the press but also recognized as serious political issues by governments [1], [4]–[6]. Many of the diseases, including late blight of potato, powdery and downy mildew of grapevine, as well as phylloxera, were due to exotic introductions from the Americas and elsewhere. These and subsequent epidemics motivated scientific investigations into crop breeding and plant disease management that developed into modern plant pathology science over the 20th century.

Nowadays, our understanding of plant pathogens and the diseases they cause greatly benefits from molecular genetics and genomics. All aspects of plant pathology, from population biology and epidemiology to mechanistic research, are impacted. The polymerase chain reaction (PCR) first enabled access to plant pathogen DNA sequences from historical specimens deposited in herbaria [7]–[9]. Historical records in combination with herbarium specimens have turned out to provide powerful tools for understanding the course of past plant epidemics. Recently, thanks to new developments in DNA sequencing technology, it has become possible to reconstruct the genomes of plant pathogens in herbaria [10], [11]. In this article, we first summarize how whole genome analysis of ancient DNA has been recently used to reconstruct the 19th-century potato-blight epidemic that rapidly spread throughout Europe and triggered the Irish potato famine. We then discuss the exciting prospects offered by the emergence of the discipline of ancient plant pathogen genomics.

## What Is Ancient DNA?

DNA retrieved from historic and prehistoric sources such as museum specimens, archaeological finds, and fossil remains is collectively known as ancient DNA (aDNA) [12]. The aDNA field goes back to the 1980s. With the invention of PCR, aDNA research came into its own, and PCR-based methods were its mainstay for 20 years. Early on, mycologists recognized the value of herbaria in storage of pathogen aDNA and used PCR to decode fragments of aDNA from dried herbarium specimens [7]–[9], [13]. In contrast to DNA extracted from fresh tissue, aDNA comes, in general, in tiny amounts, is highly fragmented, and contains chemical modifications [12]. Many of the difficulties resulting from these characteristics have been recently overcome, thanks to the advent of high-throughput sequencing and the development of new library-based retrieval of aDNA fragments without relying on direct amplification by PCR [14], [15]. Nowadays, it is possible to sequence complete genomes from organisms that went extinct tens of thousands of years ago, providing unique insight into their history and evolution. For example, the sequencing of the complete genomes of two archaic hominins (Neanderthals and Denisovans) has opened a window to the past and profoundly changed our views on human origins [16]–[18].

Such a historic perspective is also starting to arise for infectious diseases. Past epidemics and disease emergence have been reconstructed by high-throughput sequencing of genomic fragments of ancient pathogens from human skeletal remains [19]. The complete genome sequences of medieval bacterial pathogens have started to answer questions about the origin and history of infectious human diseases such as bubonic plague and leprosy [20]–[22]. Such whole genome sequence analyses of historical pathogens have recently been extended to plant pathogens [10], [11], [23]. Using herbarium material, it was possible to describe not only sequence variation in 19th-century samples of the pathogen *Phytophthora infestans*, which triggered the Irish potato famine of 1845 to 1847, but also genomic variation in its potato host. The surprisingly high quality of aDNA in these samples suggests that the millions of dried plant samples that are stored in herbaria throughout the world hold great promise for the future study of past plant epidemics.

## Using aDNA to Date Divergence Events

To understand disease dynamics, it is of particular importance to accurately date major events, such as epidemics' emergence and reemergence, and sudden changes in genetic diversity. These dated events can then be correlated with a timeline of historical and socioeconomic information. Fortunately, the genetic information obtained from aDNA sequences provides a unique opportunity to date divergence times in a phylogenetic tree. Genomes from historic samples will accumulate fewer nucleotide substitutions than modern samples, which have continued to accumulate substitutions for many more generations [18], [22]. This can be used to calculate substitution rates and subsequently divergence times using the sample dates as tip calibration points in a Bayesian phylogenetic framework [24], [25]. Herbaria samples usually contain collection date and geographic location, which makes them ideal calibration points in a phylogenetic tree [10]. Historic and modern samples that are more spread out in time yield more accurate calculations of divergence times [24].

## Potato Late Blight: From the Irish Potato Famine to Modern Epidemics

**Figure 1:**
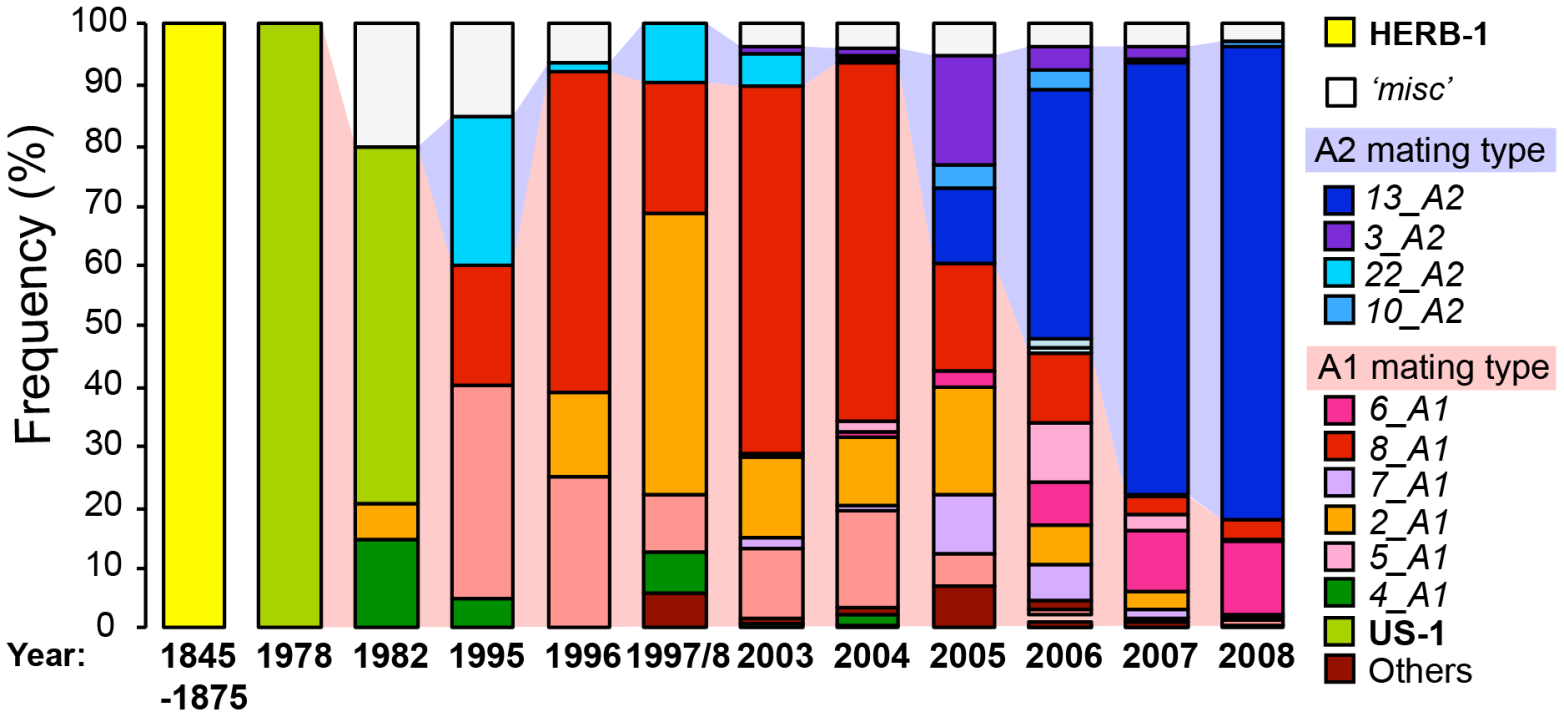
The rise and fall of *P. infestans* lineages in the British Isles. Frequency of genotypes over the years from the potato blight outbreaks in the British Isles (adapted from Cooke et al. (2012) [28]). Isolates of genotypes that occurred at a low frequency in a single year were grouped under the category termed *‘misc’*. Two mating types of *P. infestans*, A1 and A2, are necessary for sexual reproduction. The shading between the bars indicates the proportion of A1- and A2-mating-type isolates, with pink referring to the A1 mating type and blue to A2. The data of 1845–1875 and 1978 were from Yoshida et al. (2013) [10] and Fry et al. (1992) [39], respectively. A template for the figure was kindly provided by Dr. David Cooke, James Hutton Institute, Dundee [28].

The oomycete plant pathogen *P. infestans* causes late blight, the most destructive disease of potatoes and a major disease of tomatoes. Ever since the Irish potato famine in the 1840s, *P. infestans* has remained one of the more serious threats to food production, resulting in losses that add up to enough crop to feed hundreds of millions of people [26]. Similar to other oomycetes, *P. infestans* has a complex life cycle that includes both asexual and sexual phases. The ability of *P. infestans* to generate large amounts of asexual spores is a major determinant of its success as a destructive pathogen [27]. In agricultural systems, asexual reproduction is often dominant, with individual genotypes taking over large portions of the population and occasionally resulting in explosive epidemics (Figure 1) [28], [29]. Sexual reproduction is integral to the life cycle of *P. infestans* at its center of diversity in Mexico. In some agricultural systems, notably in the Netherlands and the Nordic countries, sexual reproduction is also common, generating an abundance of new pathogen genotypes [30], [31]. *P. infestans* genotypes with favorable phenotypes, such as increased virulence and spore production, can arise from pockets of sexual reproduction to become invasive when they spread to vulnerable potato production areas [28]. One example, illustrated in Figure 1, is clonal lineage 13_A2, an aggressive genotype that emerged in Europe in the early 21st century, rapidly displaced other genotypes [28], and more recently became pandemic with severe outbreaks in India [32] and China [33].

## Reconstructing the 19th-Century Potato Blight Pandemic

A widely accepted hypothesis is that *P. infestans* has originated from Toluca Valley, Mexico, where it naturally infects and coevolves with wild *Solanum* relatives of the potato [31]. Although the Spanish introduced the potato to Europe in the 16th century, the crop remained free of late blight until the mid-19th century. In 1845, *P. infestans* finally reached Europe. Potato blight was first detected in Belgium at the end of June 1845. Over the summer, the disease rapidly spread over the European continent to reach the British Isles [4]. In Ireland, socioeconomic and political circumstances coincided to turn the disease into a tragic and deadly affair—the Great Famine triggered by potato late blight killed around one million people and forced even more to emigrate.

**Figure 2:**
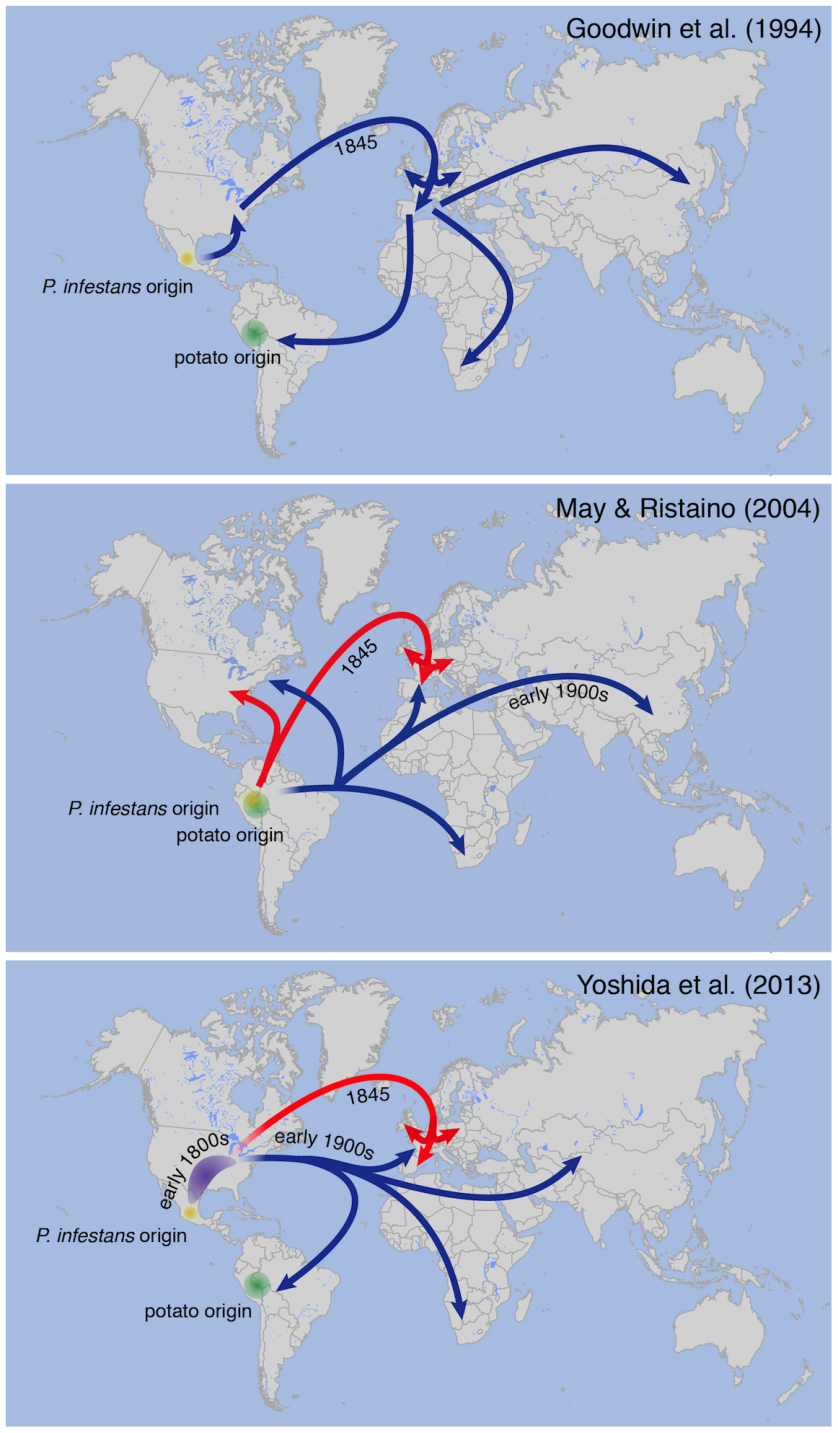
Different models proposed for 19th-century *P.* infestans pandemics. Migration paths of *P. infestans* lineages proposed by different authors [34], [8], [10]. The bottom panel of Figure 2 was adapted from Yoshida et al. (2013) [10] and reflects the most likely scenario based on the current data. The red arrows refer to the HERB-1 lineage.

The identity of the *P. infestans* strain that caused the 19th-century epidemic and its relationship to modern isolates have long been controversial topics, even though historical records suggest that the strain(s) that first caused late blight in North America in 1843 moved to Europe in 1845 [4]. Based on mitochondrial markers in modern strains, Goodwin and colleagues [34] proposed in 1994 that a clonal lineage known as US-1, which dominated the world populations until the 1970s, was a direct descendant of the strain that caused the North American and European epidemics of the 1840s (Figure 2A). Ristaino and colleagues [8], [9] subsequently used PCR analyses to monitor polymorphic nucleotide positions in mitochondrial DNA from herbarium samples. Based on the results, the authors proposed a different scenario, that the 19th-century strain was different from the US-1 lineage, and proposed that the two genotypes caused unrelated pandemics that originated in South America (Figure 2B).

**Figure 3:**
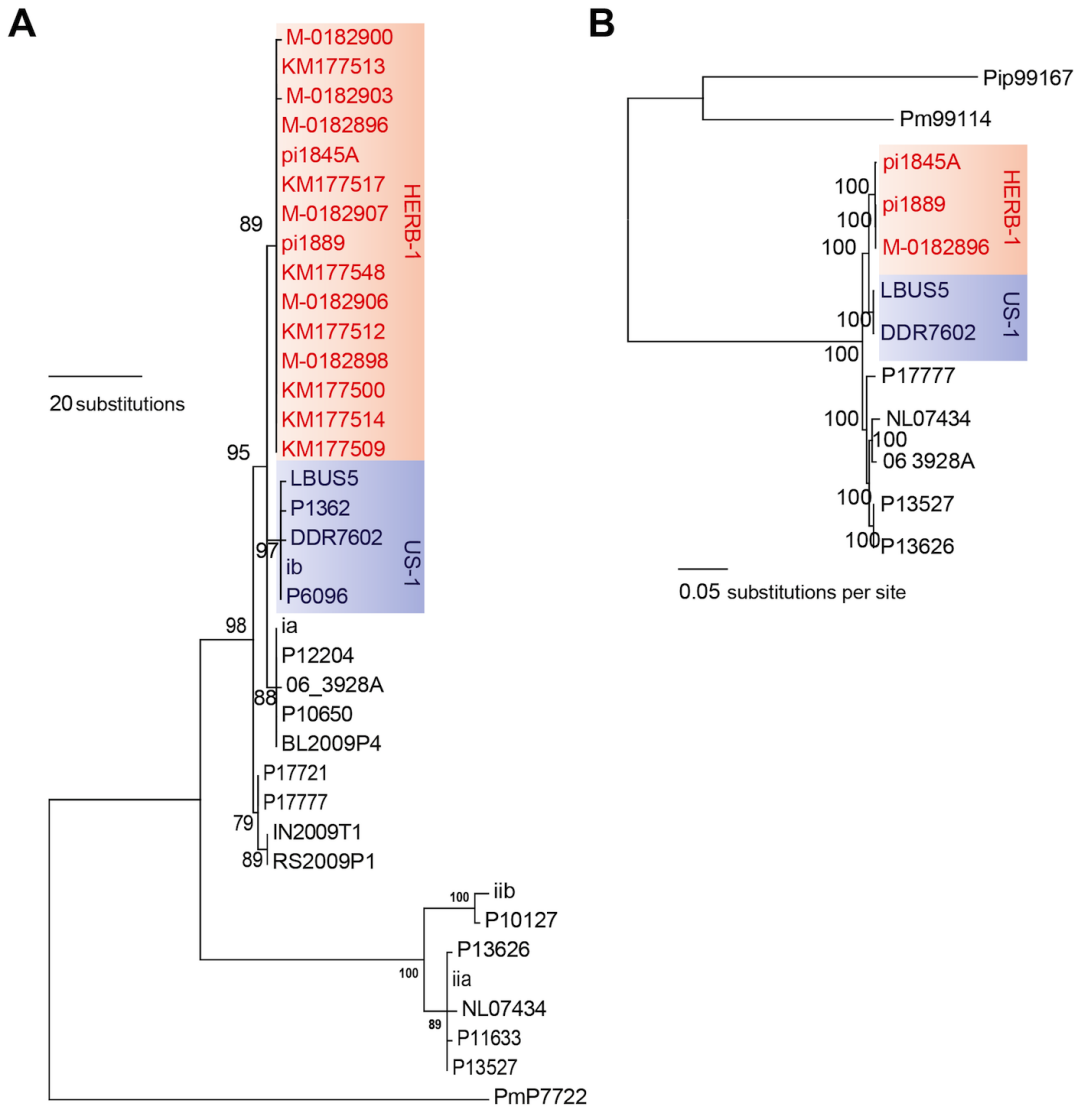
The 19th-century *P. infestans* belong to a single clonal lineage, HERB-1. (A) Maximum-parsimony tree of complete mtDNA genomes. Sites with less than 90% information were not considered, leaving 24,526 sites in the final dataset. (B) Maximum-likelihood tree based on nuclear SNPs. Only sites with 100% information were considered, leaving 2,359,452 sites in the final dataset. Numbers at branches of both trees indicate bootstrap support (100 replicates), and scales indicate genetic distance. PmP7722 and Pm99114 are *P. mirabilis*. Pip99167 is *P. ipomoeae*. These three strains were used as outgroup species. The details of the tree construction were described in Yoshida et al. (2013) [10].

To resolve these contradictory findings, two recent studies performed genome analyses of *P. infestans* aDNA obtained from 19th-century herbarium material [10], [11]. Yoshida and colleagues (2013) [10] sequenced aDNA from 11 historical strains as well as 15 modern strains to various levels of genome coverage. Their analyses revealed that the 19th-century epidemic was caused by a single genotype, HERB-1, that was widespread in Europe from 1845 to 1877. Phylogenetic analyses supported HERB-1 being distinct from the 20th-century US-1 genotype but HERB-1 and US-1 being nevertheless closely related to each other (Figure 3). Yoshida et al. proposed that the 19th- and 20th-century pandemics were caused by distinct clonal lineages, HERB-1 and US-1, but that both genotypes most likely originated from a secondary metapopulation rather than from the Mexican center of origin (bottom panel of Figure 2). This interpretation was motivated by the high degree of *P. infestans* genetic diversity and preponderance of sexual reproduction in Mexico, making it less likely that closely related genotypes would have emerged independently and successfully spread from this region. The location of the metapopulation and origin of HERB-1 remain unclear. A search for the HERB-1 genotype in the past and modern American populations of *P. infestans* will help to answer these questions.

In an independent study, Martin and colleagues [11] reported on the genomes of *P. infestans* from another five 19th-century herbarium samples. Based on the observed number of single-nucleotide polymorphisms (SNPs) between two samples collected in 1845 and 1889, they proposed an alternative scenario, with multiple clonal lineages having been introduced to and spread over Europe. This study, however, did not address directly the relationship of the 19th-century *P. infestans* sequences to the US-1 genotype. To compare the two herbarium studies [10], [11], we have estimated genetic divergence for the high-coverage genome sequences of modern and historical samples from both studies and constructed phylogenetic trees. Genetic distances among the historical samples fell within the distance between sibling isolates of two modern clonal lineages: US-1 and the South American EC-1 (Table 1). Both mitochondria and nuclear trees show that the historic samples group into one single clade (Figure 3A, 3B). Based on these results, we conclude that the historic genome sequences reported in both recent studies [10], [11] are consistent with the hypothesis that a single clonal lineage, HERB-1, caused the 19th-century potato-blight pandemic (Figure 3A, 3B).

**Table 1:** Genetic distance between individual isolates of clonal lineages HERB-1, US-1, and EC-1 of *P. infestans*.

	HERB-1 (19th century)			US-1		EC-1	
	Pi1845A^a^	M-0182896^b^	Pi1889^a^	DDR7602^b^	LBUS5^b^	P13527^b^	P13626^b^
Pi1845A							
M-0182896	**0.012** c						
Pi1889	**0.013** c	**0.008** c					
DDR7602	0.053	0.05	0.052				
LBUS5	0.054	0.05	0.052	**0.024** c			
P13527	0.056	0.054	0.056	0.064	0.064		
P13626	0.056	0.054	0.056	0.062	0.063	**0.017** c	

a Sequences from Martin et al. (2013) [11].

b Sequences from Yoshida et al. (2013) [10].

c Bold letters indicates comparisons within a clonal lineage; genetic distance was estimated based on 2,359,452 SNPs as described in Yoshida et al. (2013) [10].

## Has Modern Potato Breeding Driven HERB-1 to Extinction?

An obvious question is whether *P. infestans* HERB-1 was so devastating because it was extraordinarily virulent. Several different lines of argument suggest that this was not the case. Rather, it was the extreme blight susceptibility of the potato cultivars that were grown in the 19th century, such as the Irish Lumper potato, that was responsible for the unusual severity of the disease [4], [35]. Later on, breeding for potato-blight resistance took off at the beginning of the 20th century based on introgression of disease resistance (*R*) genes from wild relatives of the potato. Among the first *R* genes to be bred, the *R1*, *R2*, *R3a*, and *R4* genes were introduced into cultivated potatoes from the wild Mexican species *Solanum demissum* [35], [36]. These *R* genes encode immune receptors that detect specific avirulence effectors in *P. infestans* [37]. As expected from the historical records of plant breeding, HERB-1 carries isoforms of several pathogen effectors in their avirulence configuration [4], meaning that they would have triggered a resistance reaction by the *R1*, *R2*, *R3a*, and *R4* genes as well as most of the other *R* genes that are present in modern potato cultivars. This indicates that the HERB-1 genotype of *P. infestans* would probably be incapable of infecting most modern potato varieties. Indeed, HERB-1 could not be identified among the modern isolates examined to date. However, an exhaustive survey of hundreds of *P. infestans* genotypes from all corners of the globe is needed before a definitive conclusion about HERB-1 extinction is reached [38]. Nonetheless, a plausible scenario is that the HERB-1 clonal lineage was displaced by US-1 with the emergence of modern potato breeding. Genome analyses of *P. infestans* population from the first half of the 20th century will help to shed light on this question.

An important insight from the herbarium analyses is that the displacement of HERB-1 by US-1 is reminiscent of modern population dynamics (Figure 1). For example, in the British Isles, different *P. infestans* genotypes have dominated the population since the 1970s (Figure 1) [28]. Waves of *P. infestans* clonal lineages rise and fall for a variety of ecological and agricultural reasons, including breeding of disease resistance genes, chemical treatments, and climate fluctuations [28], [31], [39].

Today, with both the A1 and A2 mating types of *P. infestans* coexisting in regional pockets outside of Mexico, the trend has accelerated with a notable increase in genetic diversity compared to the period before the 1980s when only the A1 mating type occurred outside of Mexico [31], [39].

## Perspectives in Archaeogenomics of Plant Pathogens

Genome analyses of *P. infestans* aDNA have demonstrated that the potential of archaeogenomics to solve important questions in history and evolution does not apply to only humans and their pathogens but also to plant pathology. The budding field of archaeogenomics holds particular promise for plant pathogens because of the excellent preservation of genomic DNA in dried plant samples stored in herbaria. Thus, herbaria are hidden treasures, serving as unexploited archives of plant pathogen and plant genomes. Beyond the potato late blight, there are numerous interesting targets for investigation of plant pathogens. European herbaria hold over 800,000 specimens of rust fungi [40], a group of plant pathogens that are a recurring threat to world agriculture [41]. Another, well-sampled plant pathogen is the oomycete *Plasmopara viticola*, which caused epidemics of downy mildew on grapevine [5]. Mining these and other historic herbarium samples for plant pathogen genomes should inform past population and evolutionary dynamics of important plant pathogens, ultimately helping us to better prepare for future plant epidemics.
